# Identification and experimental validation of hub genes underlying depressive-like behaviors induced by chronic social defeat stress

**DOI:** 10.3389/fphar.2024.1472468

**Published:** 2024-10-14

**Authors:** Yexiang Chen, Yunhao Jiang, Xingcong Jiang, Caiyu Zhai, Yifei Wang, Chi Xu

**Affiliations:** ^1^ Department of Neurobiology and Acupuncture Research, Key Laboratory of Acupuncture and Neurology of Zhejiang Province, The Third Clinical Medical College, Zhejiang Chinese Medical University, Hangzhou, China; ^2^ Key Laboratory of Neuropharmacology and Translational Medicine of Zhejiang Province, School of Pharmaceutical Sciences, Zhejiang Chinese Medical University, Hangzhou, China; ^3^ Department of Nephrology, The First Affiliated Hospital of Zhejiang Chinese Medical University (Zhejiang Provincial Hospital of Chinese Medicine), Hangzhou, Zhejiang, China

**Keywords:** major depressive disorder (MDD), differentially expressed genes (DEGs), aspartylglucosaminidase (AGA), F-box protein 38 (FBXO38), regulator of G protein signaling 5 (RGS5), chronic social defeat stress (CSDS)

## Abstract

**Introduction:** Major depressive disorder (MDD), characterized by severe neuropsychiatric symptoms and significant cognitive deficits, continues to present both etiological and therapeutic challenges. However, the specific underlying mechanisms and therapeutic targets remain unclear.

**Methods:** We analyzed human postmortem dorsolateral prefrontal cortex (dlPFC) samples from MDD patients using datasets GSE53987 and GSE54568, identifying three key genes: AGA, FBXO38, and RGS5. To model depressive-like behavior, we employed chronic social defeat stress (CSDS) and subsequently measured the expression of AGA, FBXO38, and RGS5 in the dlPFC using qPCR and Western blot analysis following CSDS exposure.

**Results:** CSDS significantly induced depressive-like behavior, and both the protein and transcriptional expression levels of AGA, FBXO38, and RGS5 in the dlPFC of mice were markedly reduced after stress, consistent with findings from datasets GSE53987 and GSE54568.

**Conclusion:** Our research suggests that AGA, FBXO38, and RGS5 are potential biomarkers for MDD and could serve as valuable targets for MDD risk prediction.

## Introduction

Major depressive disorder (MDD) is broadly acknowledged as one of the principal urgent mental health problems, and over 264 million people of all ages are affected by this problem currently (Liu et al., 2020; Monroe And Harkness, 2022). MDD is an emotional disorder marked by a continuous sense of melancholy and/or a lack of ability to feel pleasure, accompanied by impairments in daily functioning, it is also the primary suicidal risk factor, and suicide statistics in the United States have risen by approximately 35% since 1999 (Mccarron et al., 2021; Smith, 2014). Based on the World Health Organization (WHO), depression is a primary contributor to global psychological and physical handicaps and a significant contributor to the worldwide disease burden (Mccarron et al., 2021). Although there is extensive literature on MDD clinical symptoms, the detailed cause and pathogenesis remain largely unknown. Thus, it is essential to study the etiology and development mechanisms of MDD.

The comprehensive utilization of transcriptome-wide gene expression profiling has been instrumental in uncovering MDD-associated genes, identifying disease-specific biomarkers, and predicting therapeutic efficacy. Chronic unpredictable mild stress (CUMS)-induced depressive-like behavior is associated with a significant increase in the expression of Adrald, Creb5, Itga4, and Crhr2 genes in the Ventral dorsal tegmental area (VTA), concomitant with a decrease in the expression of Tbxa2r, Tyrp1, Cplx3, and Ntf3 genes (Sun et al., 2018). CSDS-induced depressive-like behavior is characterized by a significant upregulation of Abra, Sell, and GPR35 gene expression in the prefrontal cortex (PL) (Chen Y. et al., 2023). CSDS induces depressive-like behavior via a reduction in GRP55 expression within the hippocampus (Shen et al., 2022). However, these studies are based on animal levels. Results would be more compelling if clinical samples were analyzed first, followed by validation in animal models. Nevertheless, the absence of objective assessment techniques makes decisive conclusions and treatment options for depression challenging. To augment the effectiveness of therapeutic strategies, it is imperative to establish novel biological markers that are intimately associated with depression.

Our study sought to elucidate the changes in gene transcription involved in the abnormal physiology of MDD and to identify novel diagnostic indicators. We rigorously analyzed two datasets from the GEO and identified 55 DEGs from human *postmortem* dlPFC samples in MDD. Key modules related to MDD were discerned, and through the application of RF, SVM-RFE, and LASSO algorithms, three hub genes-AGA, FBXO38, and RGS5 were identified. Subsequently, leveraging hub genes, we formulated and substantiated a prognostic nomogram for the clinical diagnosis of MDD. The diagnosticative efficacy of the three hub genes was confirmed with robust accuracy through receiver operating characteristic (ROC) curve analysis. At the same time, we utilized the CSDS model to conduct molecular biological validation of three hub genes in the dlPFC, and the results were consistent with the dataset analysis. Together, the identified triplet of crucial genes is capable of ameliorating the assessment of MDD in high-risk individuals, thereby aiding in the comprehension of the neuropsychiatric mechanisms underlying depression.

## Materials and methods

### Data processing

**FIGURE 1:**
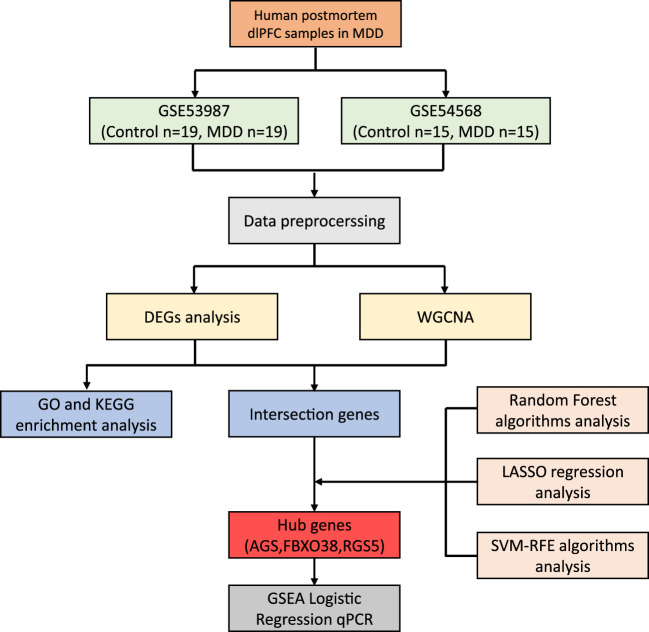
The workflow of the research.

The datasets GSE53987^1^ and GSE54568^2^, obtained from the GPL570 platform (HG-U133_Plus_2) Affymetrix Human Genome U133 Plus 2.0 Array, were sourced from the GEO database. We gathered information comprising dlPFC, with GSE53987 containing 19 pairs in normal and MDD samples, and GSE54568 including 15 pairs in normal and MDD samples. Subsequently, the R packages limma (linear models for microarray data) and sva (surrogate variable analysis) were utilized for the integration and regularization of the profiles. Probes that did not correspond to any known gene were excluded. When multiple probes matched a single gene, their mean expression was calculated. Perl (a programming language) was utilized to filter out other brain regions and disease profiles and generate dlPFC mRNA matrix files in MDD. Data normalization after processing was performed using the R package ggplot2. The diagram flow in our study is presented in Figure 1.

### Differentially expressed gene identification

The gene expression matrix of the GSE53987 and GSE54568 datasets were analyzed with the “limma” package in R to obtain DEGs between MDD and healthy specimens. Briefly, |log2 fold change (FC)| > 0.3 and a *p*-value <0.01 were established as the criteria for identifying DEGs (Davis and Meltzer, 2007; Wang et al., 2024). The ggplots package was used to create the heatmap and volcano plot (Gu et al., 2016).

### Enrichment analysis

To find out the biological significance and functions of the genes, DEGs were investigated using GO and the KEGG analysis. A *p*-value threshold below 0.05 was designated as the cutoff criterion.

### Weighted gene co-expression network analysis

We integrated and processed the data from datasets GSE53987 and GSE54568 in batches. The Weighted Gene Co-expression Network Analysis (WGCNA) package was employed to evaluate the trait-associated modules. An expression profile was used to form a topological adjacency matrix. Core modules were identified with a soft thresholding parameter set as 5 and the minimum allowed size for a module is 30. Modules were combined using a height threshold of 0.25 as a guideline (Zhang et al., 2022). Subsequently, the Pearson’s correlation test was applied to evaluate the modules, with a statistical significance is *p* < 0.05.

### RF, LASSO, and SVM-RFE model construction

Initially, predicted genes were identified by intersecting DEGs from the WGCNA hub module. Subsequently, hub genes were determined by integrating gene sets identified through the RF algorithm with the RF R package (Paul et al., 2018), the LASSO algorithm using the glmnet package (Vasquez et al., 2016), and the SVM-RFE model construction method with the e1071 package (Noble, 2006).

### Gene Set Enrichment Analysis

Gene Set Enrichment Analysis (GSEA) is a computational method used to assess the concordance of a collection of genes that are significantly enriched. The “c2.cp.kegg.v6.2.symbols.gmt” gene set was procured from the Molecular Signature Database (MSigDB), with exclusion criteria applied to enrichment sets containing the genes involved under 10 or over 200 in number. Pathways that had a normalized enrichment score (NES) above zero were deemed to be upregulated, whereas those with an NES below zero were deemed to be downregulated. The identification of the five most crucial pathways was established with a false discovery rate (FDR) of less than 0.05.

### Animals

Male C57BL6/J mice weighing 20 g ± 2 g were supplied by Shanghai Slack Laboratory Animal Liability Co., Ltd. (SCXK (Shanghai) 2017-0005) and group-housed with five mice per cage. Male CD1 mice weighing 35 g ± 5 g were purchased from Beijing Vital River Laboratory Animal Technology Co., Ltd. (SYXK (Zhejiang) 2021-0012) and housed individually for at least 7 days before use. All mice were accommodated at the Laboratory Animal Center of Zhejiang Chinese Medical University, an institution accredited by the Association for Assessment and Accreditation of Laboratory Animal Care (AAALAC). The mice were kept in a controlled environment with a temperature of 24°C ± 1°C and humidity between 50% and 60%. The mice were kept under a 12-h light-dark cycle and were provided with unlimited access to food and water. All experimental protocols were sanctioned by the Animal Ethics Committee of Zhejiang Chinese Medical University (approval number: IACUC-20220328-09) and adhered to the National Institute of Health Guide for the Care and Use of Laboratory Animals (NIH Publications No. 80-23).

### Chronic social defeat stress (CSDS)

We employed a CSDS paradigm simulating human depressive-like behavior to establish a mouse model exhibiting depressive-like behaviors. The CSDS model was developed based on our previously published research (Chen Y. et al., 2023; Wang et al., 2023; Zan et al., 2021). Briefly, in a consecutive 3-day period, aggressive CD1 mice were assessed for aggressive behavior with a 3-min assessment each day. Subsequently, the selected aggressive CD1 mice were utilized for a 10-min daily stress regimen applied to the experimental C57BL/6J mice, spanning a consecutive 10-day period. Following each aggressive encounter, the C57BL/6J mice were housed adjacent to the C57BL/6J mice, separated by a partition with perforations, which allows sensory contact without physical contact. At the end of CSDS, depressive-like behavior tests such as social interaction test (SIT), tail suspension test (TST), forced swim test (FST), and sucrose preference test (SPT) were assessed.

### Social interaction test (SIT)

SIT was employed to assess the sociability of mice. The protocol was conducted within an open-field apparatus (40 cm × 40 cm × 40 cm), with a rectangular wire cage (14 cm × 8 cm × 24 cm) installed at one end of the testing area. Twice 3-min social interaction zone (14 cm × 26 cm) in the open-field arena will be recorded by video tracking software (Shanghai Jiliang Software Technology). The first session will feature an empty rectangular mesh enclosure, while the second session will include a CD1 mouse within the mesh enclosure. The social interaction ratio (SIR) was calculated as the time spent in the interaction zone in the second session divided by the time spent in the interaction zone in the first session (Chen Y. et al., 2023).

### Tail suspension test (TST) and forced swim test (FST)

TST and FST were used to measure behavioral despair in mice. In TST, mice were suspended by adhering the last 2 cm of their tail tips to an object for 6 min. In FST, mice were placed in a water-filled plastic cylinder (16 cm diameter; 26 cm height) maintained at 25°C ± 1°C and were forced to swim for 6 min. The immobility time in the TST and FST was recorded for the last 4 min, with the initial 2 min allocated for the mice to acclimate to the environment (Chen Y. et al., 2023).

### Sucrose preference test (SPT)

The SPT was conducted to assess anhedonia in mice. Mice were acclimated for 3 days with two bottles of water to correct for any position bias. Subsequently, the mice were subjected to a 24-h water deprivation period, followed by unrestricted access to two bottles for 2 hours, one bottle containing water and the other containing 2% sucrose solution. The weights of the water and sucrose solution bottles were recorded before and after a 2-h interval. The SPT score was calculated as follows: [sucrose intake/(sucrose intake + water intake) × 100%] (Chen Y. et al., 2023).

### Locomotor activity test (LAT)

The LAT was utilized to assess the spontaneous activity of mice unaffected by CSDS. Mice were positioned in an open-field apparatus (40 cm × 40 cm × 40 cm) equipped with an infrared video camera for 10 min. The total distance travelled by mice was recorded by the Animal Behavior Analysis System (Shanghai Jiliang Software Technology Co., Ltd.).

### qPCR validation

After behavioral assessments, the dorsolateral prefrontal cortex (dlPFC) was collected for total mRNA extraction with TRIzol reagent (Invitrogen, 15596026CN, United States). 500 ng of mRNA was reverse transcribed employing the PrimeScript™ RT reagent Kit with gDNA Eraser (Takara, RR047A, Japan), and the quantity of mRNA was assessed via real-time PCR using LightCycler^®^ 480 SYBR^®^ Green Ⅰ Master (Roche, 04887352001, Germany) on the LightCycler^®^ 480 Real-Time PCR System (Roche, Germany). Three hub gene primers were bought from Sangon Biotech (China) and primer information is shown in Table 1. Relative gene expression was determined through the methodology of 2^−ΔΔCt^, with the results normalized to the levels of GAPDH.

**TABLE 1:** Primers of AGA, FBXO38, and RGS5 genes.

Primers information (mouse)		
Gene name	Forward primers	Reverse primers
AGA	GCG​TGG​TGG​ACA​TTG​CTA​TCT​GG	CCA​TCA​CAC​TGC​TCC​TTC​TCA​CAC
FBXO38	TCG​TCC​AGA​CCT​ACA​AGC​AGG​AG	TTC​CAC​TCT​CGG​CTA​CCA​GTT​CTC
RGS5	TGT​GTA​AGG​GAC​TGG​CAG​CTC​TG	GCA​GGC​TTC​TCT​GGC​TTC​TCA​TTG

### Western blot validation

After behavioral assessments, the dlPFC was collected and lysed in RIPA (P0013B, Beyotime, China) containing phosphatase and protease inhibitor cocktail. Equal amounts of protein were subjected to 10% SDS-PAGE, followed by transferring onto PVDF transfer membranes (IPVH00010, Merk, United States). The membranes were incubated overnight at 4°C with primary antibodies of RGS5 (1:500, A7015, ABclonal, China), FBXO38 (1:1,000, 83509-6-RR, proteintech, China), AGA (1:2000, 83442-6-RR, proteintech, China) and β-actin (1:80,000, AC026, ABclonal, China) diluted by Western Rapid Kit (L00884, GenScript, China). After incubated with HRP-conjugated Goat anti-Rabbit IgG (H + L) (1:5,000, AS003, ABclonal) for 2 h at room temperature, the signals were detected by Fluor Chem R(protein simple, biotech, United States) with ECL chemiluminescence kit (BL523A, Biosharp, China) and analyzed by ImageJ.

### Statistical analysis

The R software (version 4.1.3) was utilized to inspect the data, and the Wilcoxon test was applied to determine group variations, with *p* < 0.05 considered as the criterion for statistical significance.

## Results

### Identification of DEGs in MDD and healthy control samples

**FIGURE 2:**
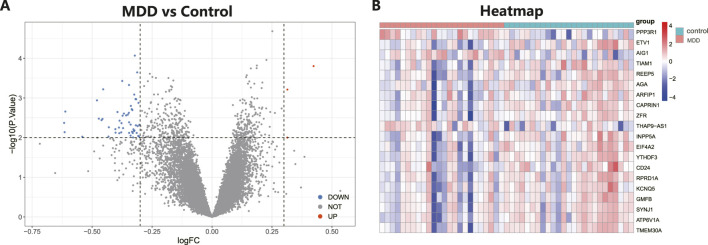
Differential expressed genes (DEGs) analysis in MDD and healthy control samples. **(A)** The volcano plot showed differential expressed genes (DEGs) in MDD and normal samples (Blue, gene downregulated; Red, gene upregulated; Gray, gene not variated). **(B)** The heat map displayed the DEGs of control and MDD samples.

The workflow of the research is shown in Figure 1. Our study utilized two rounds of microarray profiling, GSE53987 and GSE54568, to investigate DEGs. The combined expression matrix revealed 55 DEGs, consisting of 52 genes that were downregulated and 3 genes that were upregulated. The volcano plot and heatmap of the DEGs are depicted in Figures 2A, B, respectively.

### Functional enrichment analysis

**FIGURE 3:**
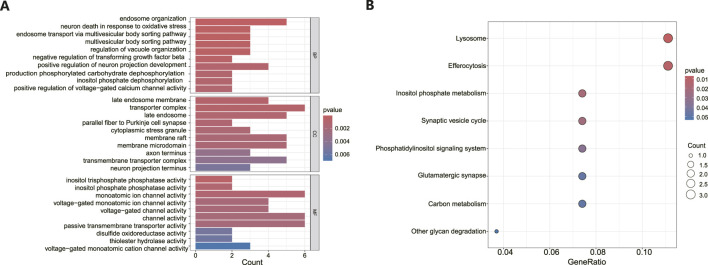
Functional enrichment analysis of DEGs in MDD and healthy control samples. **(A)** The bar graph displays the top 10 highest-ranking genes in terms of molecular functions (MF), biological processes (BP), and cellular components (CC) annotations for DEGs, as determined by GO functional enrichment analysis. **(B)** A bar diagram represented the KEGG pathways enrichment analysis results for DEGs.

Following the identification of DEGs, functional enrichment analysis was conducted using the GO database. Figure 3A presents the top 10 GO terms with the lowest *p*-values and the most pronounced enrichment for each respective GO category histogram. DEGs were predominantly enriched in biological processes (BP) such as endosome organization and positive regulation of neuron projection development. In terms of cellular components (CC), the DEGs were mainly richer in structures including transporter complexes, late endosomes, membrane rafts, and membrane microdomains. Regarding molecular functions (MF), the DEGs showed enrichment in activities such as monatomic ion channel activity, passive transmembrane transporter activity, and channel activity. Based on the KEGG pathway enrichment analysis, the enrichment factor and *p*-values were also utilized. The analysis discovered that DEGs were dominantly concentrated in lysosome and efferocytosis pathways, depicted in Figure 3B.

### Overlap between MDD-associated genes with DEGs

**FIGURE 4:**
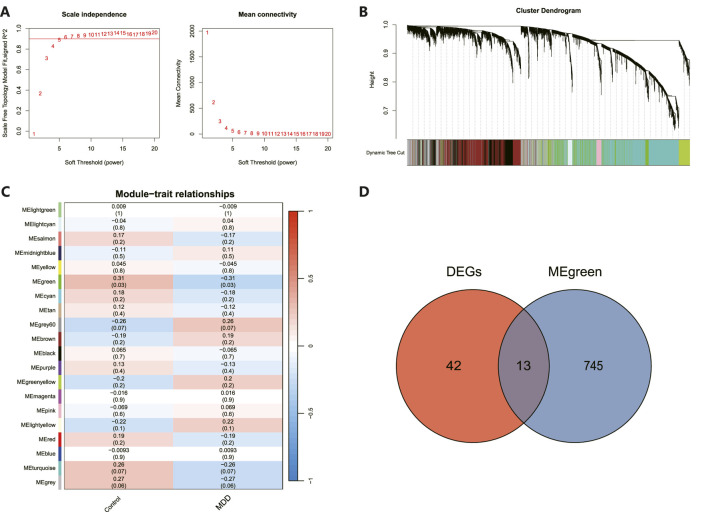
WGCNA revealed pivotal modules. **(A)** Scale-free fit indicators and mean connectivity for different soft threshold capabilities. **(B)** Aggregation of clusters of DEGs based on topological overlap dissimilarity. **(C)** Module-trait showed the relationships between control and MDD samples. Each row corresponds to a list of modules, while each column corresponds to a clinical characteristic. The associated correlation is presented in the first line of each cell, and the *P*-value is provided in the second line. **(D)** Intersecting genes are shown in the Venn diagram.

As illustrated in Figure 4A, an architecture of a network that exhibits scale-free properties and utilizes a gentle threshold of 5 was developed, with an R^2^ = 0.9 indicating a strong correlation. We next computed the module eigengenes, which depict the collective gene expression levels within each module, and organized them based on their connections. As depicted in Figure 4B, twenty modules were identified. A single module showed a correlation with MDD (green; correlation = −0.31, *p*-value = 0.03). The 758 genes linked to MDD within this module were preserved for additional study, as shown in Figure 4C. Ultimately, 13 genes were identified to intersect between the DEGs and the chosen genes within module MEgreen, as depicted in Figure 4D.

### Hub gene identification and diagnostic evaluation

**FIGURE 5:**
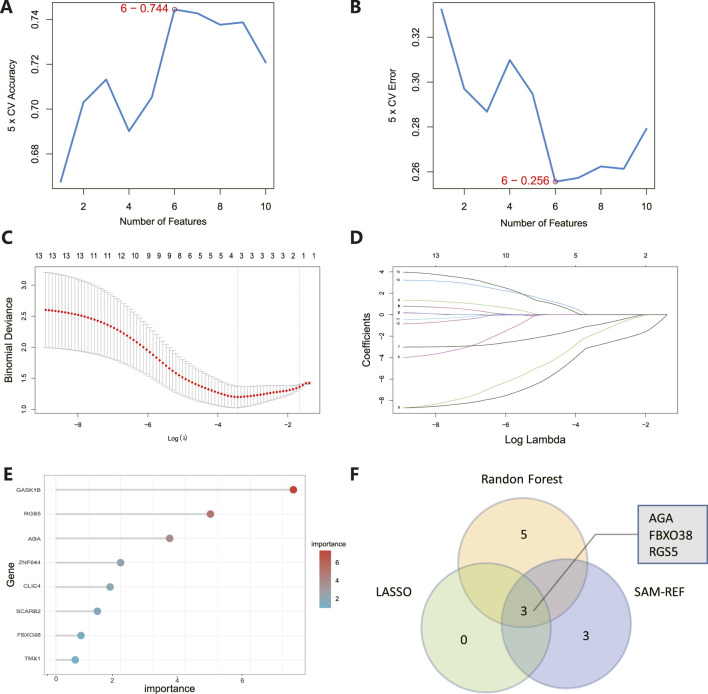
Identification of hub genes. **(A)** SVM-RFE analysis with an accuracy of 0.744 discovered six gene signatures. **(B)** SVM-RFE analysis with an Error of 0.256 identified six gene signatures. **(C)** LASSO regression analysis was performed to select the optimal tuning parameter log (Lambda) for cross-validation. **(D)** Candidate genes were selected by LASSO coefficient profiles. **(E)** The predictive accuracy of the RF model. **(F)** Venn diagram illustrating three key hub genes of methodologies including SVM-RFE, RF, and LASSO.

**FIGURE 6:**
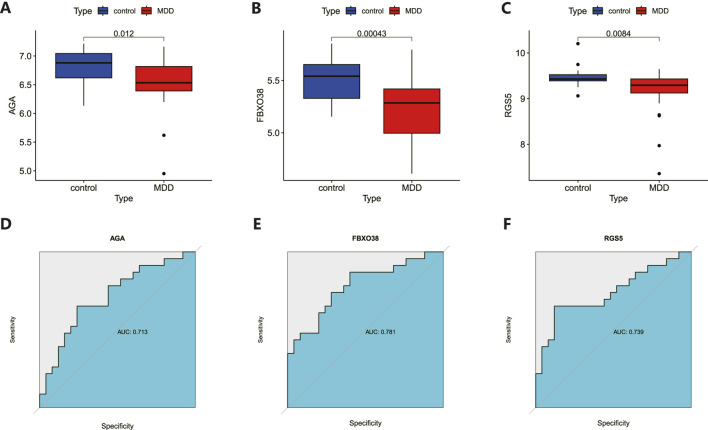
Analysis and diagnostic evaluation of hub gene expression. **(A–C)** Expression of three hub genes (AGA, FBXO38, RGS5) of dlPFC in MDD and control groups. AGA, FBXO38, and RGS5 were significantly reduced in MDD compared with control samples. **(D–F)** ROC curve for assessing the predictive accuracy of three AGA, FBXO38, and RGS5.

The 8 candidate genes were examined using RF, LASSO, and SVM-RFE methods to pinpoint gene signatures. We developed 6 gene signatures using SVM-RFE, achieving a precision of 0.744, and an error of 0.256, as depicted in Figures 5A, B. LASSO regression analysis revealed 3 distinct gene signatures, as shown in Figures 5C, D. The RF method identified 8 genes with importance scores greater than two, as displayed in Figure 5E. To establish a robust gene signature for MDD, we pinpointed genes that intersected across three methods, culminating in the identification of three pivotal genes: AGA, FBXO38, and RGS5, highlighted in Figure 5F. Compared to control, AGA, FBXO38, and RGS5 were notably decreased in MDD samples, as illustrated in Figures 6A–C. We conducted a further evaluation of the diagnostic significance of the three hub genes: AGA, FBXO38, and RGS5. The AUC values for the hub genes in MDD and healthy samples were as follows: AGA had an AUC of 0.713, FBXO38 had an AUC of 0.781, and RGS5 had an AUC of 0.739, as demonstrated in Figures 6D–F. These findings suggest that the three hub genes could have substantial diagnostic significance for MDD.

### Gene set enrichment of the hub genes

**FIGURE 7:**
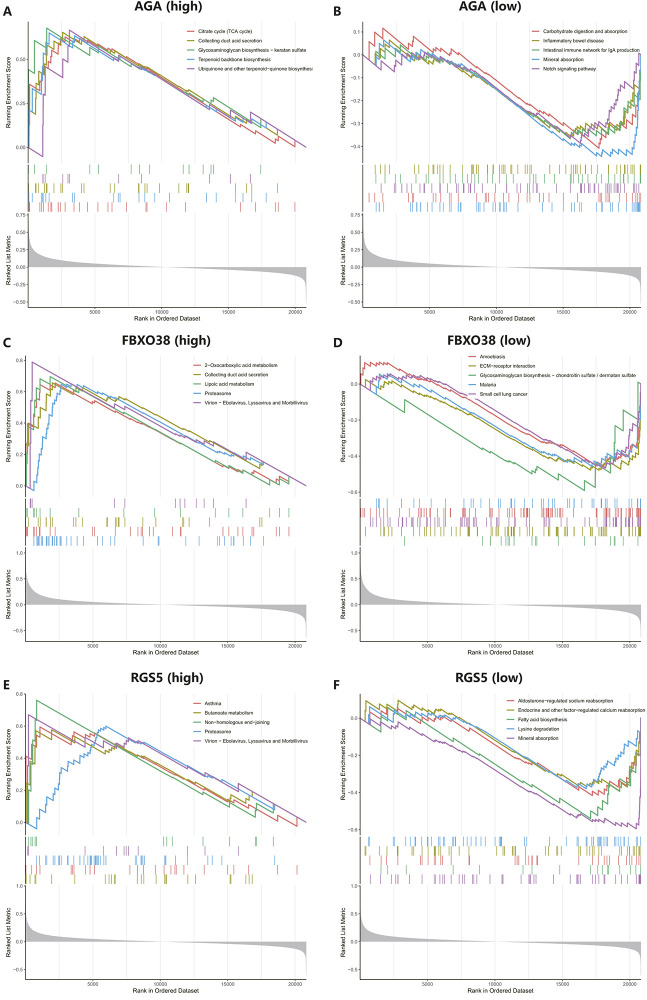
Performing gene set enrichment analysis (GSEA) analysis of three hub genes. **(A, B)** High and low expression of top 5 GSEA enrichment in AGA. **(C, D)** The top 5 GSEA enrichments exhibited both high and low expression levels about FBXO38. **(E–F)** The top 5 GSEA enrichments showed variable expression levels, including both high and low expression to RGS5.

We conducted GSEA to better understand the potential functions of AGA, FBXO38, and RGS5. The genes that exhibit high expression levels belong to the three key hub genes were mainly rich in collecting duct acid secretion, proteasome, virion-ebolavirus, and morbillivirus, while genes that fall into the categories of low expression with the three hub genes were mainly enriched in mineral absorption, as shown in Figure 7.

### Depressive-like behaviors induced by CSDS

**FIGURE 8:**
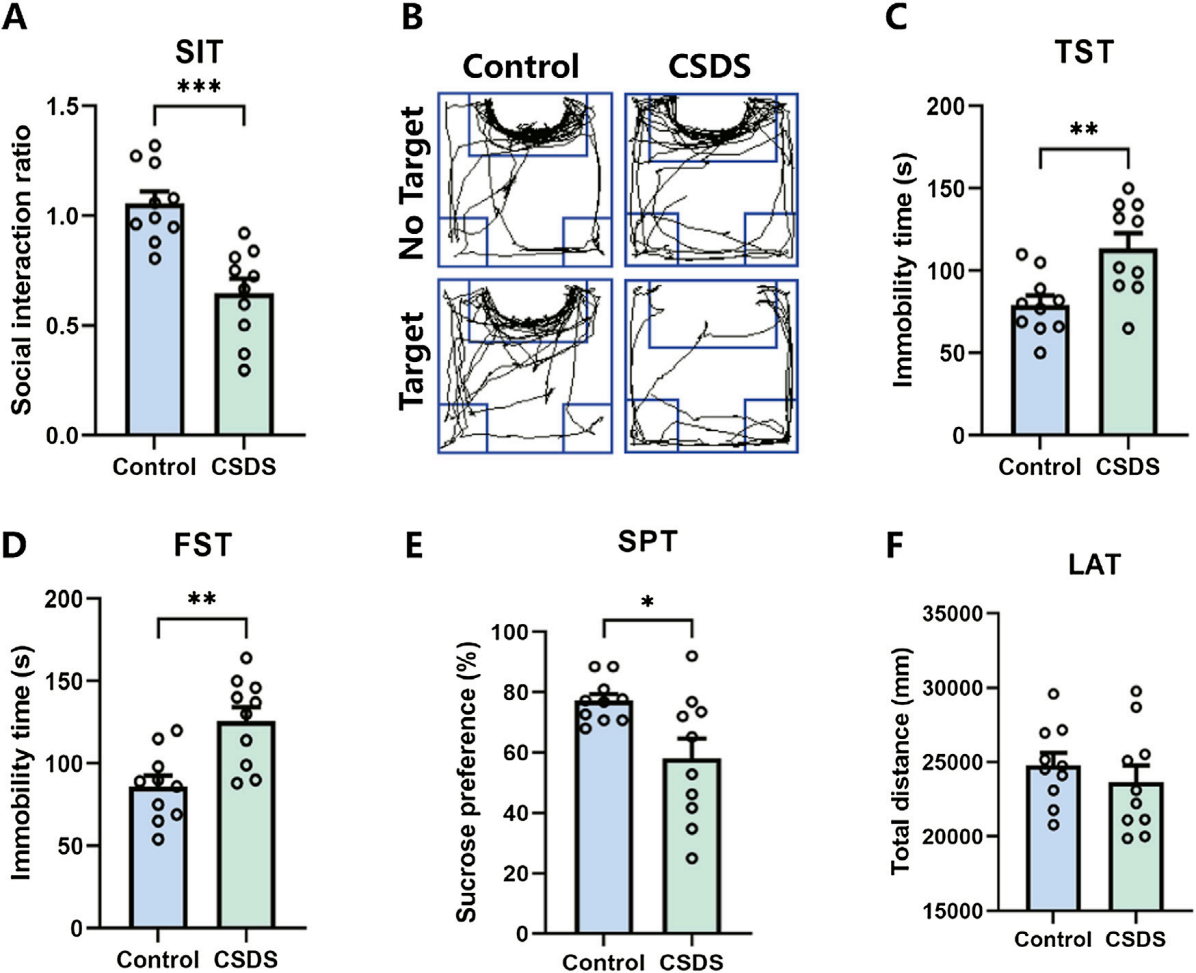
CSDS elicited depressive-like behaviors without affecting locomotor activity. **(A, B)** CSDS decreased the social interaction ratio in the social interaction test (SIT) (A. n = 10, t (18) = 4.801, *p* = 0.0001; B. Representative exploration tracks in the SIT). **(C–D)** CSDS increased the immobility time in the tail suspension test (TST) (C. n = 10, t (18) = 3.257, *p* = 0.0044) and forced swim test (FST) (D. n = 10, t (18) = 3.692, *p* = 0.0017). **(E)** CSDS reduced sucrose consumption in the sucrose preference test (SPT) (n = 10, t (18) = 2.712, *p* = 0.0143). **(F)** No significant variance was observed in the locomotor activity test (LAT) (n = 10, t (18) = 0.8153, *p* = 0.4255). Student’s t-test. Data expressed as Mean ± SEM. *p < 0.05, **p < 0.01, ***p < 0.001.

It is proposed that chronic social defeat stress (CSDS) can effectively elicit depressive-like behaviors in mice. After the CSDS program, depressive-like behavior was performed. Research has revealed that mice subjected to CSDS displayed notable social withdrawal, feelings of despair, and a lack of pleasure, as indicated by a reduction of social interaction ratio in the social interaction test (SIT), an extension of immobility periods in the tail suspension test (TST) and forced swim test (FST), and a decrease in sucrose consumption in the sucrose preference test (SPT), as shown in Figures 8A–E. No significant difference in locomotor activity test (LAT) was detected between the control and CSDS mice, as shown in Figure 8F. These findings suggest that the CSDS paradigm elicits depression-like phenotypes in mice models.

### qRT-PCR and Western blot validation of hub gene

**FIGURE 9:**
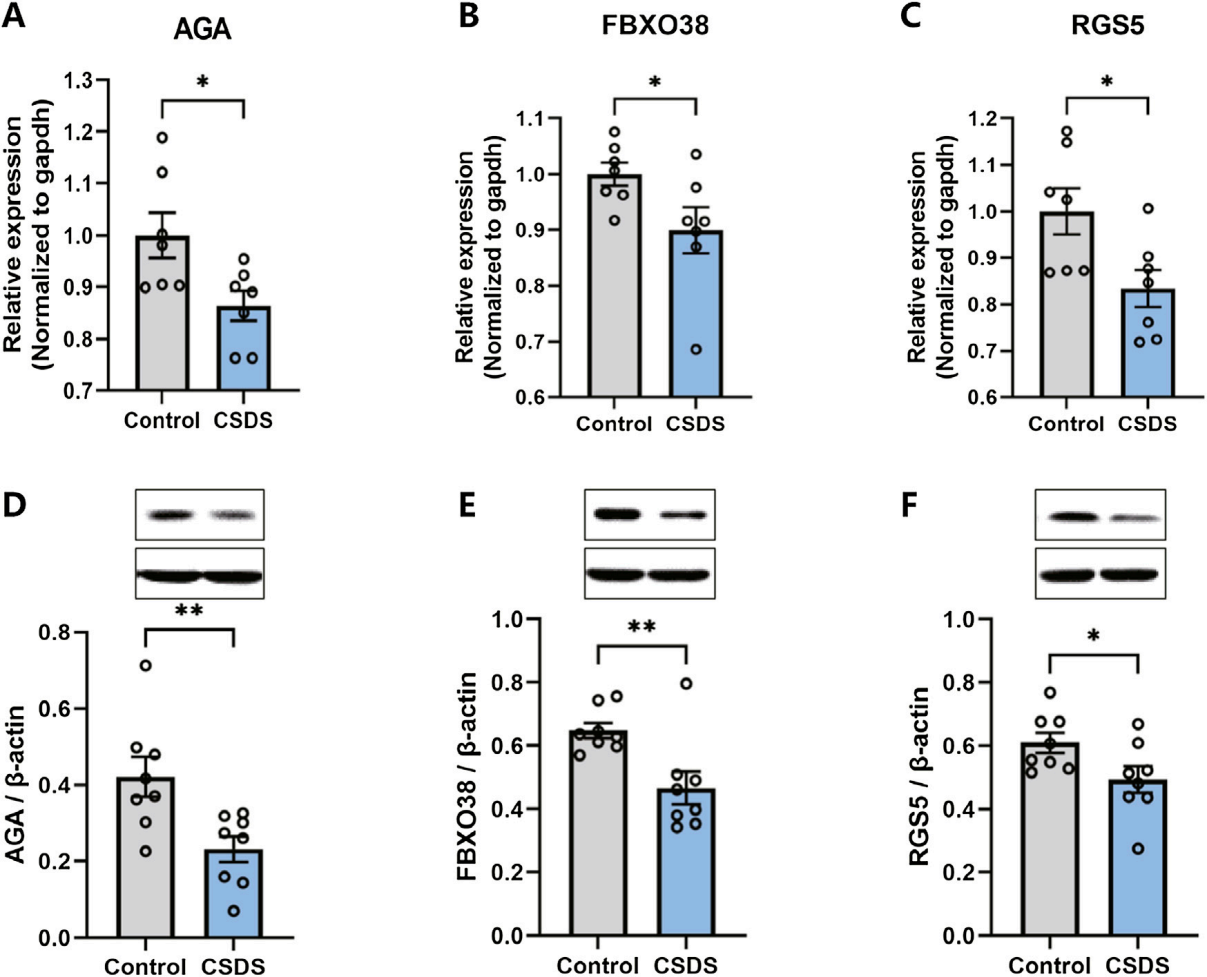
The expression of AGA, FBXO38, and RGS5 decreased after CSDS. **(A–C)** CSDS reduced the gene expression of AGA (A. n = 7, t (12) = 2.622, *p* = 0.0223), FBXO38 (B. n = 7, t (12) = 2.184, *p* = 0.0495), and RGS5 (C. n = 7, t (18) = 2.605, *p* = 0.023), detected by PCR. **(D–F)** CSDS reduced the protein expression of AGA (D. n = 8, t (14) = 3.05, *p* = 0.0087), FBXO38 (E. n = 8, t (14) = 3.19, *p* = 0.0065), and RGS5 (F. n = 8, t (14) = 2.20, *p* = 0.0451), detected by Western blot. Student’s t-test. Data expressed as Mean ± SEM. *p < 0.05, **p < 0.01.

Following the behavioral assessments, qRT-PCR and Western blot were conducted to confirm the alterations in the expression of the hub genes in the dlPFC of mice subjected to CSDS compared to the control group. As depicted in Figure 9, the expression of AGA, FBXO38, and RGS5 were found to be downregulated in the CSDS group compared with the control group.

## Discussion

MDD is a chronic, recurrent psychiatric disorder and heterogeneous condition, characterized by recurrent periods of remission and exacerbation. The clinical manifestations of MDD encompass emotional dysregulation, loss of interest, sleep disturbances, and difficulty concentrating or making decisions, which are attributed to disturbances in the immunological, metabolic, and endocrine systems (Kessler et al., 2005; Cipriani et al., 2018; Søvold et al., 2021; Battle, 2013). The underlying mechanisms of MDD remain incompletely elucidated. Traditionally, diminished activity of monoaminergic neurotransmitters-serotonin, norepinephrine, dopamine, or a combination thereof-has been implicated in the pathophysiology, with effective antidepressant treatments presumed to rectify these functional deficiencies (Park and Zarate, 2019). Research indicates that MDD is strongly associated with epigenetics, which refers to the processes that impact gene expression and translation without involving changes in the DNA sequence, including DNA methylation (DNAm), microRNAs (miRNAs), and histone modifications (Penner-Goeke and Binder, 2019). Hence, elucidating the pivotal pathways and genetic signatures associated with MDD may facilitate risk assessment, elucidation of pathogenesis, and the development of individualized therapeutic strategies.

In this study, we performed an extensive analysis of MDD utilizing dorsolateral prefrontal cortex (dlPFC) samples, which was identified as a pivotal region associated with MDD pathology. At the molecular level, depression is delineated by impaired neuroplasticity, encompassing neuronal atrophy and synaptic dysfunction in the medial prefrontal cortex (mPFC) (Price and Duman, 2020). The mPFC not only maintains significant functional connectivity with other brain regions such as the ventral tegmental area (VTA), claustrum (CLA), amygdala (BLA), mediodorsal thalamus (MD), nucleus accumbens (NAC), ventral hippocampus (vHPC), anterior ventral bed nucleus of the stria terminalis (avBNST) but also plays a crucial role at the molecular level (Wang et al., 2023; Bagot et al., 2015; Lv et al., 2024; Chaudhury et al., 2013; Chen JY. et al., 2023; Vouimba and Maroun, 2011; Tang et al., 2020; Li et al., 2024). However, the etiology of this dysfunction and its precise contributions remain obscure.

Genes like Est1, Cacna1c, and Dcc, which are linked to sensitivity to stress and psychiatric conditions, are also associated with MDD (Torres-Berrío et al., 2017). β-catenin, identified as a pivotal gene in the network, has been shown to modulate social stress, and its NAc-selective knockout in mice resulted in increased vulnerability to chronic stress, while its overexpression in the NAc improved stress resilience (Penner-Goeke and Binder, 2019). Studies have detected the expression of four genes encoding COX-2, MPO, iNOS, and secretory phospholipase A2 type IIA, namely PTGS2, MPO, NOS2A, and PLA2GA, and found that they are all elevated in patients with depression, highlighting the interrelationship between MDD and mRNA (Gałecki et al., 2012). Genome-wide association studies (GWAS) have revealed associations for numerous psychiatric conditions, including schizophrenia, autism, and bipolar disorder (Schizophrenia Working Group of the Psychiatric Genomics Consortium, 2014; Gaugler et al., 2014; Nurnberger et al., 2014). Studies have identified 317 upregulated genes in MDD patients using GWAS and candidate gene approaches, with these genes enriched in synaptic transmission pathways and protein-protein interaction networks, including the same calcium-signaling genes (CACNA1B, CACNA1E) as those found in the Psychiatric Genomics Consortium (PGC) study (CACNA1C and CACNB2) (Hori et al., 2016; Ripke et al., 2013). Transcriptomic research has indicated that the neural cell-adhesion molecule L-1-like protein (CHL-1) could serve as a potential biomarker for depression, as discovered in two early genome-wide transcription studies (Morag et al., 2011; Oved et al., 2012). In a study involving 463 patients and 459 controls, Mostafavi and colleagues identified a significant link between MDD and the IFN-α/-β signaling pathway, mediated by the overexpression of IFN-stimulated gene factor 3 (ISGF3)-induced genes (Mostafavi et al., 2014). And gene expression in major depressive disorder has also been researched by R (Jansen et al., 2016).

Due to substantial progress in bioinformatics, microarray data can now effectively uncover hub genes, interaction networks, and pathways relevant to MDD. In this study, the DEGs were predominantly associated with neurofunctional activities. Utilizing bioinformatics approaches, this pioneering study has identified three pivotal genes (AGA, FBXO38, and RGS5) intricately associated with MDD. The deficiency of aspartylglucosaminidase (AGA) results in aspartylglucosaminuria (AGU), a lysosomal storage disorder characterized by glycoasparagine accumulation (Goodspeed et al., 2021; Banning et al., 2023). Additional neurological signs might encompass seizures, impaired balance and coordination, and advancing brain atrophy as adults (Goodspeed et al., 1993). Children with aspartylglucosaminuria are often characterized by heightened activity levels and may satisfy the diagnostic criteria for attention-deficit/hyperactivity disorder. From adolescence onward, individuals may experience restlessness or anxiety symptoms, progressing in adulthood to potential episodes of psychosis or the development of apathy, which can result in social withdrawal and autism spectrum disorder (Goodspeed et al., 2021; Arvio and Mononen, 2016). F-box protein 38 (FBXO38) belongs to the F-box family of proteins, characterized by the presence of an F-box motif at its amino terminus (Jin et al., 2004). FBXO38 serves as a recognized coactivator of the transcription factor Krüppel-like factor 7 (KLF7), crucial in governing genes essential for neuronal axon outgrowth and regeneration (Sumner et al., 2013; Mcconnell and Yang, 2010). FBXO38, exhibits broad expression across the developing nervous system, encompassing motor neurons (Smaldone et al., 2004; Laub et al., 2001). FBXO38 is associated with the group of neurological disorders, with its Online Mendelian Inheritance in Man (OMIM) phenotype characterized as Neuronopathy, distal hereditary motor, a type of lysosomal storage disorder (Jauss et al., 2022). FBXO38 was also discovered in rare neurogenetic diseases and neuromuscular diseases (Grunseich et al., 2021; Megarbane et al., 2022). Regulators of G protein signaling (RGS) proteins are pivotal transduction molecules that critically regulate heterotrimeric G proteins (Jules et al., 2015). The regulator of G protein signaling 5 (RGS5) is one member of RGS. RGS5, acting as a GTPase-activating protein (GAP) specific to the Gαi subunit, exerts negative regulation on G protein-coupled receptor (GPCR) signaling and was observed in primary mouse cortical neurons (Liu et al., 2017; Bondjers et al., 2003). RGS5 is involved in neurological disorders, including diseases of Huntington’s (HTT), Parkinson’s disease (PD), Alzheimer’s disease (AD), stroke, and so on. According to Robert Carlsson, RGS5 has an important response of brain pericytes to hypoxia (Carlsson et al., 2023). It has been reported that RGS5 increased in the striatum of pericytes in mouse model of AD (Padel et al., 2016; Berger et al., 2005). The absence of RGS5 impairs pericyte-associated maintenance of the blood-brain barrier (BBB) during stroke, highlighting RGS5 as a crucial target for neurovascular protection (Özen et al., 2018; Roth et al., 2019; Özen et al., 2014).

Correlation analysis revealed a strong positive correlation among the three hub genes, suggesting their pivotal role in MDD pathology. In the current study, two MDD datasets are combined, the three hub genes-AGA, FBXO38, and RGS5-exhibited AUCs exceeding 0.7, indicating their robust diagnostic potential. We also utilized the CSDS-induced depressive-like behavior model, which can effectively simulate depression. Following behavioral assessments, transcription and protein levels of AGA, FBXO38, and RGS5 in the dlPFC were measured, with the results consistent with analyses from two datasets, GSE53987 and GSE54568. Approximately 80% of genes are co-expressed in peripheral blood and brain tissues, and exhibit similar regulation at the mRNA level; the analysis of mRNA changes in brain tissues may also be changed within peripheral blood is now an integrated approach for discovering biomarkers of mental health (Hepgul et al., 2013). Together, the hub genes of AGA, FBXO38, and RGS5 hold significant promise as diagnostic markers and therapeutic targets for MDD.

An overview of our study reveals some remaining shortcomings. Firstly, the etiology of depression is multifaceted, and our study has only validated depressive-like behaviors induced by CSDS and the expression of three hub genes implicated in CSDS-induced depression. Secondly, we have solely confirmed the transcription levels of these three hub genes in the dlPFC post-CSDS using qPCR and Western blot; due to constraints in pharmacological resources, we did not further investigate depression intervention through gene agonists or inhibitors. Thirdly, there is a need for further integration with clinical samples for in-depth analysis.

## Conclusion

Utilizing bioinformatics approaches, a gene signature comprising AGA, FBXO38, and RGS5, closely linked to MDD, was initially identified. AGA, FBXO38, and RGS5 genes also exhibited significant downregulation in the CSDS-induced depressive-like behavior. They may serve as critical targets for depression and offer valuable insights for clinical research on the disorder.

## Data Availability

The original contributions presented in the study are included in the article, further inquiries can be directed to the corresponding authors.

## Footnotes

1
https://www.ncbi.nlm.nih.gov/geo/query/acc.cgi?acc=GSE53987

2
https://www.ncbi.nlm.nih.gov/geo/query/acc.cgi?acc=GSE54568

## References

[B2] Arvio M. Mononen I. (2016). Aspartylglycosaminuria: a review. Orphanet J. rare Dis. 11 (1), 162. 10.1186/s13023-016-0544-6 27906067 PMC5134220

[B3] Bagot R. C. Parise E. M. PeñA C. J. Zhang H. X. Maze I. Chaudhury D. (2015). Ventral hippocampal afferents to the nucleus accumbens regulate susceptibility to depression. Nat. Commun. 6, 7062. 10.1038/ncomms8062 25952660 PMC4430111

[B4] Banning A. Laine M. Tikkanen R. (2023). Validation of aspartylglucosaminidase activity assay for human serum samples: establishment of a biomarker for diagnostics and clinical studies. Int. J. Mol. Sci. 24 (6), 5722. 10.3390/ijms24065722 36982794 PMC10059667

[B5] Battle D. E. (2013). Diagnostic and statistical manual of mental disorders (DSM). CoDAS 25 (2), 191–192. 10.1590/s2317-17822013000200017 24413388

[B6] Berger M. Bergers G. Arnold B. Hämmerling G. J. Ganss R. (2005). Regulator of G-protein signaling-5 induction in pericytes coincides with active vessel remodeling during neovascularization. Blood 105 (3), 1094–1101. 10.1182/blood-2004-06-2315 15459006

[B7] Bondjers C. KaléN M. HellströM M. Scheidl S. J. Abramsson A. Renner O. (2003). Transcription profiling of platelet-derived growth factor-B-deficient mouse embryos identifies RGS5 as a novel marker for pericytes and vascular smooth muscle cells. Am. J. pathology 162 (3), 721–729. 10.1016/S0002-9440(10)63868-0 PMC1868109 12598306

[B8] Carlsson R. EnströM A. Paul G. (2023). Molecular regulation of the response of brain pericytes to hypoxia. Int. J. Mol. Sci. 24 (6), 5671. 10.3390/ijms24065671 36982744 PMC10053233

[B9] Chaudhury D. Walsh J. J. Friedman A. K. Juarez B. Ku S. M. Koo J. W. (2013). Rapid regulation of depression-related behaviours by control of midbrain dopamine neurons. Nature 493 (7433), 532–536. 10.1038/nature11713 23235832 PMC3554860

[B10] Chen J. Y. Wu K. Guo M. M. Song W. Huang S. T. Zhang Y. M. (2023b). The PrL(Glu)→avBNST(GABA) circuit rapidly modulates depression-like behaviors in male mice. iScience 26 (10), 107878. 10.1016/j.isci.2023.107878 37810240 PMC10551841

[B11] Chen Y. Yao S. Y. Shu X. Wang Y. J. Liu J. G. (2023a). Changes in mRNA and miRNA expression in the prelimbic cortex related to depression-like syndrome induced by chronic social defeat stress in mice. Behav. brain Res. 438, 114211. 10.1016/j.bbr.2022.114211 36368442

[B12] Cipriani A. Furukawa T. A. Salanti G. Chaimani A. Atkinson L. Z. Ogawa Y. (2018). Comparative efficacy and acceptability of 21 antidepressant drugs for the acute treatment of adults with major depressive disorder: a systematic review and network meta-analysis. Lancet London, Engl. 391 (10128), 1357–1366. 10.1016/S0140-6736(17)32802-7 PMC5889788 29477251

[B13] Davis S. Meltzer P. S. (2007). GEOquery: a bridge between the gene expression Omnibus (GEO) and BioConductor. Bioinforma. Oxf. Engl. 23 (14), 1846–1847. 10.1093/bioinformatics/btm254 17496320

[B14] Gałecki P. Gałecka E. Maes M. Chamielec M. Orzechowska A. Bobińska K. (2012). The expression of genes encoding for COX-2, MPO, iNOS, and sPLA2-IIA in patients with recurrent depressive disorder. J. Affect. Disord. 138 (3), 360–366. 10.1016/j.jad.2012.01.016 22331023

[B15] Gaugler T. Klei L. Sanders S. J. Bodea C. A. Goldberg A. P. Lee A. B. (2014). Most genetic risk for autism resides with common variation. Nat. Genet. 46 (8), 881–885. 10.1038/ng.3039 25038753 PMC4137411

[B16] Goodspeed K. Chen X. Tchan M. Aspartylglucosaminuria M. Adam M. P. Feldman J. (1993) “University of Washington, Seattle copyright © 1993-2024,” in GeneReviews(®). University of Washington, Seattle: GeneReviews is a registered trademark of the University of Washington, Seattle. All rights reserved.

[B17] Goodspeed K. Feng C. Laine M. Lund T. C. (2021). Aspartylglucosaminuria: clinical presentation and potential therapies. J. child neurology 36 (5), 403–414. 10.1177/0883073820980904 33439067

[B18] Grunseich C. Sarkar N. Lu J. Owen M. Schindler A. Calabresi P. A. (2021). Improving the efficacy of exome sequencing at a quaternary care referral centre: novel mutations, clinical presentations and diagnostic challenges in rare neurogenetic diseases. J. neurology, Neurosurg. psychiatry 92 (11), 1186–1196. 10.1136/jnnp-2020-325437 PMC8522445 34103343

[B19] Gu Z. Eils R. Schlesner M. (2016). Complex heatmaps reveal patterns and correlations in multidimensional genomic data. Bioinforma. Oxf. Engl. 32 (18), 2847–2849. 10.1093/bioinformatics/btw313 27207943

[B20] Hepgul N. Cattaneo A. Zunszain P. A. Pariante C. M. (2013). Depression pathogenesis and treatment: what can we learn from blood mRNA expression? BMC Med. 11, 28. 10.1186/1741-7015-11-28 23384232 PMC3606439

[B21] Hori H. Sasayama D. Teraishi T. Yamamoto N. Nakamura S. Ota M. (2016). Blood-based gene expression signatures of medication-free outpatients with major depressive disorder: integrative genome-wide and candidate gene analyses. Sci. Rep. 6, 18776. 10.1038/srep18776 26728011 PMC4700430

[B22] Jansen R. Penninx B. W. Madar V. Xia K. Milaneschi Y. Hottenga J. J. (2016). Gene expression in major depressive disorder. Mol. psychiatry 21 (3), 444–447. 10.1038/mp.2015.94 26100536

[B23] Jauss R. T. SchließKE S. Abou Jamra R. (2022). Routine diagnostics confirm novel neurodevelopmental disorders. Genes 13 (12), 2305. 10.3390/genes13122305 36553572 PMC9778535

[B24] Jin J. Cardozo T. Lovering R. C. Elledge S. J. Pagano M. Harper J. W. (2004). Systematic analysis and nomenclature of mammalian F-box proteins. Genes and Dev. 18 (21), 2573–2580. 10.1101/gad.1255304 15520277 PMC525538

[B25] Jules J. Yang S. Chen W. Li Y. P. (2015). Role of regulators of G protein signaling proteins in bone physiology and pathophysiology. Prog. Mol. Biol. Transl. Sci. 133, 47–75. 10.1016/bs.pmbts.2015.02.002 26123302 PMC4817727

[B26] Kessler R. C. Berglund P. Demler O. Jin R. Merikangas K. R. Walters E. E. (2005). Lifetime prevalence and age-of-onset distributions of DSM-IV disorders in the national comorbidity survey replication. Archives general psychiatry 62 (6), 593–602. 10.1001/archpsyc.62.6.593 15939837

[B27] Laub F. Aldabe R. Friedrich V. JR. Ohnishi S. Yoshida T. Ramirez F. (2001). Developmental expression of mouse Krüppel-like transcription factor KLF7 suggests a potential role in neurogenesis. Dev. Biol. 233 (2), 305–318. 10.1006/dbio.2001.0243 11336497

[B28] Li F. Zheng X. Wang H. Meng L. Chen M. Hui Y. (2024). Mediodorsal thalamus projection to medial prefrontal cortical mediates social defeat stress-induced depression-like behaviors. Neuropsychopharmacol. official Publ. Am. Coll. Neuropsychopharmacol. 49 (8), 1318–1329. 10.1038/s41386-024-01829-y PMC11224337 38438592

[B29] Liu C. Hu Q. Jing J. Zhang Y. Jin J. Zhang L. (2017). Regulator of G protein signaling 5 (RGS5) inhibits sonic hedgehog function in mouse cortical neurons. Mol. Cell. Neurosci. 83, 65–73. 10.1016/j.mcn.2017.06.005 28684360

[B30] Liu Q. He H. Yang J. Feng X. Zhao F. Lyu J. (2020). Changes in the global burden of depression from 1990 to 2017: findings from the global burden of disease study. J. psychiatric Res. 126, 134–140. 10.1016/j.jpsychires.2019.08.002 31439359

[B31] Lv S. S. Lv X. J. Cai Y. Q. Hou X. Y. Zhang Z. Z. Wang G. H. (2024). Corticotropin-releasing hormone neurons control trigeminal neuralgia-induced anxiodepression via a hippocampus-to-prefrontal circuit. Sci. Adv. 10 (3), eadj4196. 10.1126/sciadv.adj4196 38241377 PMC10798562

[B32] Mccarron R. M. Shapiro B. Rawles J. Luo J. (2021). Depression. Ann. Intern. Med. 174 (5), Itc65–itc80. 10.7326/AITC202105180 33971098

[B33] Mcconnell B. B. Yang V. W. (2010). Mammalian Krüppel-like factors in health and diseases. Physiol. Rev. 90 (4), 1337–1381. 10.1152/physrev.00058.2009 20959618 PMC2975554

[B34] Megarbane A. Bizzari S. Deepthi A. Sabbagh S. Mansour H. Chouery E. (2022). A 20-year clinical and genetic neuromuscular cohort analysis in Lebanon: an international effort. J. Neuromuscul. Dis. 9 (1), 193–210. 10.3233/JND-210652 34602496 PMC8842757

[B35] Monroe S. M. Harkness K. L. (2022). Major depression and its recurrences: life course matters. Annu. Rev. Clin. Psychol. 18, 329–357. 10.1146/annurev-clinpsy-072220-021440 35216520

[B36] Morag A. Pasmanik-Chor M. Oron-Karni V. Rehavi M. Stingl J. C. Gurwitz D. (2011). Genome-wide expression profiling of human lymphoblastoid cell lines identifies CHL1 as a putative SSRI antidepressant response biomarker. Pharmacogenomics 12 (2), 171–184. 10.2217/pgs.10.185 21332311

[B37] Mostafavi S. Battle A. Zhu X. Potash J. B. Weissman M. M. Shi J. (2014). Type I interferon signaling genes in recurrent major depression: increased expression detected by whole-blood RNA sequencing. Mol. psychiatry 19 (12), 1267–1274. 10.1038/mp.2013.161 24296977 PMC5404932

[B38] Noble W. S. (2006). What is a support vector machine? Nat. Biotechnol. 24 (12), 1565–1567. 10.1038/nbt1206-1565 17160063

[B39] Nurnberger J. I. JR. Koller D L. Jung J. Edenberg H. J. Foroud T. Guella I. (2014). Identification of pathways for bipolar disorder: a meta-analysis. JAMA psychiatry 71 (6), 657–664. 10.1001/jamapsychiatry.2014.176 24718920 PMC4523227

[B40] Oved K. Morag A. Pasmanik-Chor M. Oron-Karni V. Shomron N. Rehavi M. (2012). Genome-wide miRNA expression profiling of human lymphoblastoid cell lines identifies tentative SSRI antidepressant response biomarkers. Pharmacogenomics 13 (10), 1129–1139. 10.2217/pgs.12.93 22909203

[B41] Özen I. Deierborg T. Miharada K. Padel T. Englund E. Genové G. (2014). Brain pericytes acquire a microglial phenotype after stroke. Acta neuropathol. 128 (3), 381–396. 10.1007/s00401-014-1295-x 24848101 PMC4131168

[B42] Özen I. Roth M. Barbariga M. Gaceb A. Deierborg T. Genové G. (2018). Loss of regulator of G-protein signaling 5 leads to neurovascular protection in stroke. Stroke 49 (9), 2182–2190. 10.1161/STROKEAHA.118.020124 30354999 PMC6116795

[B43] Padel T. Özen I. Boix J. Barbariga M. Gaceb A. Roth M. (2016). Platelet-derived growth factor-BB has neurorestorative effects and modulates the pericyte response in a partial 6-hydroxydopamine lesion mouse model of Parkinson's disease. Neurobiol. Dis. 94, 95–105. 10.1016/j.nbd.2016.06.002 27288154

[B44] Park L. T. Zarate C. A. (2019). Depression in the primary care setting. N. Engl. J. Med. 380 (6), 559–568. 10.1056/NEJMcp1712493 30726688 PMC6727965

[B45] Paul A. Mukherjee D. P. Das P. Gangopadhyay A. Chintha A. R. Kundu S. (2018). Improved random forest for classification. IEEE Trans. image Process. a Publ. IEEE Signal Process. Soc. 27 (8), 4012–4024. 10.1109/TIP.2018.2834830 29993742

[B46] Penner-Goeke S. Binder E. B. (2019). Epigenetics and depression. Dialogues Clin. Neurosci. 21 (4), 397–405. 10.31887/DCNS.2019.21.4/ebinder 31949407 PMC6952745

[B47] Price R. B. Duman R. (2020). Neuroplasticity in cognitive and psychological mechanisms of depression: an integrative model. Mol. psychiatry 25 (3), 530–543. 10.1038/s41380-019-0615-x 31801966 PMC7047599

[B48] Ripke S. Wray N. R. Lewis C. M. Hamilton S. P. Weissman M. M. Breen G. (2013). A mega-analysis of genome-wide association studies for major depressive disorder. Mol. psychiatry 18 (4), 497–511. 10.1038/mp.2012.21 22472876 PMC3837431

[B49] Roth M. Gaceb A. EnströM A. Padel T. Genové G. Özen I. (2019). Regulator of G-protein signaling 5 regulates the shift from perivascular to parenchymal pericytes in the chronic phase after stroke. FASEB J. 33 (8), 8990–8998. 10.1096/fj.201900153R 31039042 PMC6662981

[B1] Pantelish C. Papadimitriou G. N. Papiol S. Parkhomenko E. Pato M. T. Paunio T. (2014). Biological insights from 108 schizophrenia-associated genetic loci Nature 511 (7510), 421–427. 10.1038/nature13595 25056061 PMC4112379

[B50] Shen S. Y. Yu R. Li W. Liang L. F. Han Q. Q. Huang H. J. (2022). The neuroprotective effects of GPR55 against hippocampal neuroinflammation and impaired adult neurogenesis in CSDS mice. Neurobiol. Dis. 169, 105743. 10.1016/j.nbd.2022.105743 35490927

[B51] Smaldone S. Laub F. Else C. Dragomir C. Ramirez F. (2004). Identification of MoKA, a novel F-box protein that modulates Krüppel-like transcription factor 7 activity. Mol. Cell. Biol. 24 (3), 1058–1069. 10.1128/mcb.24.3.1058-1069.2004 14729953 PMC321422

[B52] Smith K. (2014). Mental health: a world of depression. Nature 515 (7526), 181. 10.1038/515180a 25391942

[B53] SøVOLD L. E. Naslund J. A. Kousoulis A. A. Saxena S. Qoronfleh M. W. Grobler C. (2021). Prioritizing the mental health and well-being of healthcare workers: an urgent global public health priority. Front. public health 9, 679397. 10.3389/fpubh.2021.679397 34026720 PMC8137852

[B54] Sumner C. J. D'Ydewalle C. Wooley J. Fawcett K. A. Hernandez D. Gardiner A. R. (2013). A dominant mutation in FBXO38 causes distal spinal muscular atrophy with calf predominance. Am. J. Hum. Genet. 93 (5), 976–983. 10.1016/j.ajhg.2013.10.006 24207122 PMC3824115

[B55] Sun X. Song Z. Si Y. Wang J. H. (2018). microRNA and mRNA profiles in ventral tegmental area relevant to stress-induced depression and resilience. Prog. neuro-psychopharmacology and Biol. psychiatry 86, 150–165. 10.1016/j.pnpbp.2018.05.023 29864451

[B56] Tang J. Lu L. Wang Q. Liu H. Xue W. Zhou T. (2020). Crocin reverses depression-like behavior in Parkinson disease mice via VTA-mPFC pathway. Mol. Neurobiol. 57 (7), 3158–3170. 10.1007/s12035-020-01941-2 32495180

[B57] Torres-BerríO A. Lopez J. P. Bagot R. C. Nouel D. Dal Bo G. Cuesta S. (2017). DCC confers susceptibility to depression-like behaviors in humans and mice and is regulated by miR-218. Biol. psychiatry 81 (4), 306–315. 10.1016/j.biopsych.2016.08.017 27773352 PMC5239724

[B58] Vasquez M. M. Hu C. Roe D. J. Chen Z. Halonen M. Guerra S. (2016). Least absolute shrinkage and selection operator type methods for the identification of serum biomarkers of overweight and obesity: simulation and application. BMC Med. Res. Methodol. 16 (1), 154. 10.1186/s12874-016-0254-8 27842498 PMC5109787

[B59] Vouimba R. M. Maroun M. (2011). Learning-induced changes in mPFC-BLA connections after fear conditioning, extinction, and reinstatement of fear. Neuropsychopharmacol. official Publ. Am. Coll. Neuropsychopharmacol. 36 (11), 2276–2285. 10.1038/npp.2011.115 PMC3176564 21750582

[B60] Wang Y. J. Zan G. Y. Xu C. Li X. P. Shu X. Yao S. Y. (2023). The claustrum-prelimbic cortex circuit through dynorphin/κ-opioid receptor signaling underlies depression-like behaviors associated with social stress etiology. Nat. Commun. 14 (1), 7903. 10.1038/s41467-023-43636-x 38036497 PMC10689794

[B61] Wang H. Cheng W. Hu P. Ling T. Hu C. Chen Y. (2024). Integrative analysis identifies oxidative stress biomarkers in non-alcoholic fatty liver disease via machine learning and weighted gene co-expression network analysis. Front. Immunol. 15, 1335112. 10.3389/fimmu.2024.1335112 38476236 PMC10927810

[B62] Zan G. Y. Wang Y. J. Li X. P. Fang J. F. Yao S. Y. Du J. Y. (2021). Amygdalar κ-opioid receptor-dependent upregulating glutamate transporter 1 mediates depressive-like behaviors of opioid abstinence. Cell Rep. 37 (5), 109913. 10.1016/j.celrep.2021.109913 34731618

[B63] Zhang C. Dong N. Xu S. Ma H. Cheng M. (2022). Identification of hub genes and construction of diagnostic nomogram model in schizophrenia. Front. aging Neurosci. 14, 1032917. 10.3389/fnagi.2022.1032917 36313022 PMC9614240

